# The Online Support System for Mental Health Problems After the Earthquake: A Prompt Response from The Psychiatric Association of Turkey

**DOI:** 10.1192/j.eurpsy.2024.172

**Published:** 2024-08-27

**Authors:** I. G. Yilmaz-Karaman, B. R. Erim, H. Karaş, E. Mutlu

**Affiliations:** ^1^Psychiatry, Eskişehir Osmangazi University, Eskişehir; ^2^Psychiatry, Private practise, Istanbul; ^3^Psychiatry, Hacettepe University, Ankara, Türkiye

## Abstract

**Introduction:**

Previous research demonstrated that disasters have adverse mental health outcomes. Preventive mental health interventions in the golden hours/days after a disaster may reduce psychological harm by getting in the way of emerging mental disorders or alleviating existing ones. The widespread use of smartphones and broad internet access enabled remote mental health interventions during disasters. After the 2023 Turkey earthquake, observing the mental health burden of the earthquake, The Psychiatric Association of Turkey conducted an online platform for psychological first aid. The aim was to unite volunteer psychiatrists and disaster survivors.

**Objectives:**

This study presents the organization of an online mental health service during a disaster while reporting the characteristics of the service users.

**Methods:**

The Psychiatric Association of Turkey called its members to volunteer for an Online Support System for Mental Problems After the Earthquake. The group regularly united to set standards of care. An action plan and algorithm were set up for applicants with acute suicidal, homicidal risk, or active psychotic symptoms in collaboration with local institutions and field volunteers. Volunteer psychiatrists were asked to collect information on the applicant’s sociodemographic characteristics, disaster experience, and mental health status.

**Results:**

Volunteer psychiatrists collected data on 180 applications. Most cases applied for themselves (%95.5), and some asked for advice to care for their relatives (%4.5). Earthquake survivors have the highest psychopathology rate (%64.9), followed by healthcare workers and search and rescue personnel (%61.1). Figure 1 summarizes the subgroups of the service users. The earthquake survivors group had a mean age of 34.45, % and 76.1 of them were female. Only %15.7 of them had lower educational levels than high school. %75 cases reached safe places on the 5th day of the disaster. Applicants reported discrimination and aggression after the catastrophe, related to experiencing mental health problems, owning a pet, looting, and being seen as less traumatized since their relatives are alive.

**Image:**

**Figure:**
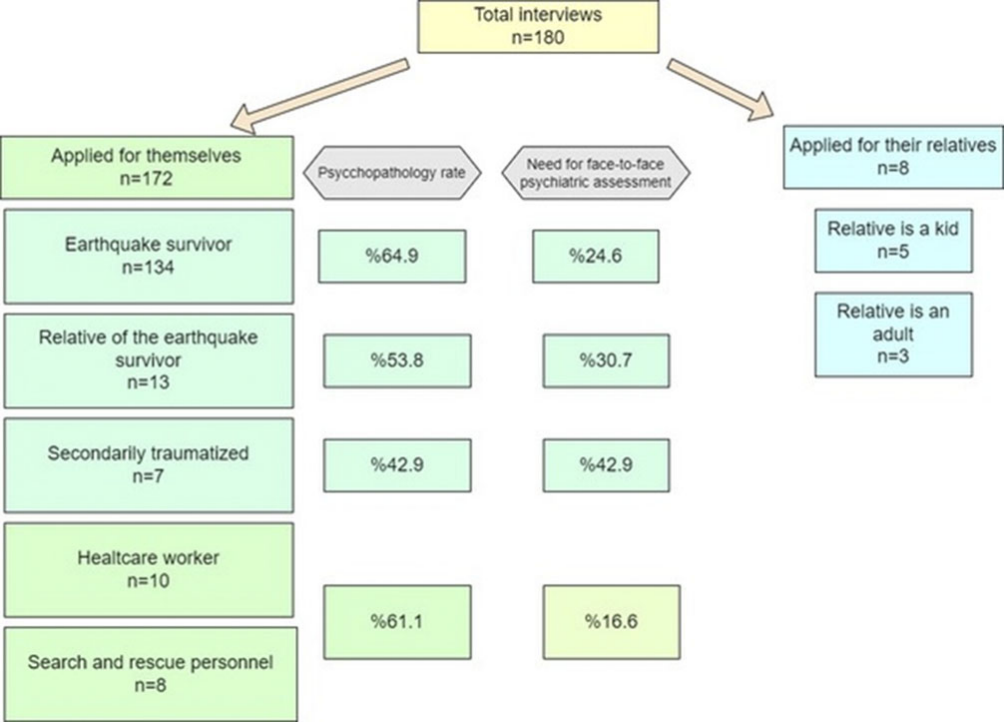

**Conclusions:**

The present experience of the Psychiatric Association of Turkey is an example of a fast and collaborative response to a disaster regarding mental health. Our results also represent the characteristics of online mental health service users during a disaster.

**Disclosure of Interest:**

None Declared

